# From surveillance to pathogenesis: characterization of genotype V of chicken infectious anemia virus

**DOI:** 10.3389/fvets.2025.1710392

**Published:** 2025-11-18

**Authors:** Zetao Su, Mei Leng, Zhiqiang Wu, Wenjing Chen, Shenghua Yang, Xuesong Li, Guanming Huo, Lijuan Yin, Jianping Qin, Wencheng Lin

**Affiliations:** 1 College of Animal Science, South China Agricultural University, Guangzhou, China; 2 College of Veterinary Medicine, South China Agricultural University, Guangzhou, China; 3 Yunfu Branch, Guangdong Laboratory for Lingnan Modern Agriculture, Yunfu, Guangdong, China; 4 Wen’s Foodstuffs Group Co., Ltd., Yunfu, Guangdong, China

**Keywords:** chicken infectious anemia virus, surveillance, genetic diversity, pathogenicity, genotype V

## Abstract

**Introduction:**

Chicken infectious anemia virus (CIAV) is a globally distributed immunosuppressive pathogen that causes substantial economic losses in the poultry industry.

**Methods:**

From 2023 to 2024, 408 clinical samples were collected from diseased chickens to investigate the molecular epidemiology and genetic diversity of CIAV strains circulating in southern China. A representative genotype V strain WSFL24 was further characterized by complete genome sequencing and pathogenicity evaluation using specific-pathogen-free (SPF) chicks.

**Results:**

Among the collected samples, 153 (37.5%) tested positive for CIAV. A total of 21 CIAV isolates were isolated and classified the isolates into genotype IIIa (13 isolates) and V (8 isolates). The genotype V strain WSFL24 possessed 31 amino acid substitutions in VP1, including virulence-associated residues (Q139, Q144, and Q394). Moreover, WSFL24 caused 60% mortality, severe anemia (hematocrit <27%), pronounced thymic atrophy, elevated viral loads in lymphoid tissues and cloacal swabs, and distinct histopathological lesions compared with the attenuated reference strain Cux-1 (genotype IIIb).

**Discussion:**

These results demonstrate the emergence and enhanced virulence of genotype V CIAV strains in southern China. The findings emphasize the need for continuous molecular surveillance and the development of updated vaccines to control evolving virulent genotypes.

## 1 Introduction

Chicken infectious anemia virus (CIAV) is a circovirus, classified within the genus *Gyrovirus* of the family *Anelloviridae* in 2015. The causative agent remains the sole member of this genus and is the smallest animal virus (1, 2). Virions are non-enveloped, with an average diameter of 23–26.5 nm. Viral genome consists of a single-stranded, negative-sense, circular DNA molecule of 2,298 or 2,319 bp (3). Since its initial identification in Japan in 1979, CIAV has become globally distributed, including in Asia, Europe, and the Americas (4, 5). In Asia, CIAV infections have been reported in China, Japan, and South Korea, with high infection rates across diverse geographical locations and chicken flock types (6–10). Since it was first reported in China, CIAV has spread across most of the country’s provinces (11). Although CIAV exists as a single serotype, pathogenicity varies significantly among isolates.

CIAV can transmit horizontally through the respiratory tract and digestive tract, causing subclinical infections that impair immune competence in chickens. Furthermore, CIAV-infected breeder hens can vertically transmit the virus to their progeny via the egg, causing systemic organ atrophy, increased mortality, and subcutaneous wing hemorrhages in the offspring flocks (12–14). To prevent CIAV, breeders administer a live attenuated virus vaccination to flocks aged between 9 and 15 weeks. Vertical transmission of CIAV can be reduced by maternal anti-CIAV antibodies in chickens (15). CIAV-infected chickens become immunosuppressed, increasing susceptibility to concurrent infections. These mixed infections further compromise the host immune system and elevate mortality (16, 17). Additionally, CIAV infection reduces vaccine efficacy (e.g., against Marek’s disease and Newcastle disease) and may enhance the pathogenicity of attenuated vaccines (18–20).

The CIAV genome contains three partially overlapping open reading frames (ORFs) that encode the VP1, VP2, and VP3 proteins (21). The VP1 protein, a capsid protein of approximately 52 kDa, is the only structural protein found on free CIAV virions (22). The *vp1* gene shows significant variation among CIAV isolates, primarily determining viral virulence. Therefore, CIAV genotyping primarily relies on *vp1* gene sequence analysis (7, 23). The VP2 protein, approximately 24 kDa in size, functions as a scaffolding protein facilitating the correct assembly of the VP1 protein into viral particles and mediates host cell binding. It may also disrupt cell signaling pathways, suppressing interferon responses and exacerbating immunosuppression (24). ORF3 encodes the 14 kD VP3 protein (Apoptin). VP3/Apoptin induces apoptosis, particularly in thymus and bone marrow T-cell precursors (25). This causes lymphoid organs (thymus and bursa of Fabricius) atrophy, consequently impairs antiviral immunity (26).

One-day-old chicks infected with CIAV develop severe anemia after 14 and 16 days infection, characterized by: (1) hematocrit levels < 20%, (2) yellow bone marrow transformation, and (3) marked atrophy of the thymus and bursa of Fabricius. Some virulent strains can cause lethal Monoinfection (27). CIAV-induced immunosuppression enhances host susceptibility to secondary infections, significantly elevating mortality rates (13). Additionally, symptoms including growth retardation and immunosuppression resulting from CIAV infection contribute to approximately 18.5% net income losses (28). Therefore, underscoring the imperative for early CIAV control is crucial. This study aimed to characterize the molecular diversity of CIAV strains in southern China (2023–2024) and to evaluate the pathogenicity of a novel genotype V strain, thereby providing valuable insights for the development of effective control strategies.

## 2 Materials and methods

### 2.1 Ethics statement

This study was approved by the Animal Care Committee of South China Agricultural University (approval ID: SYXK-2024-0136). All study procedures and animal care activities were conducted per the recommendations in the Guide for the Care and Use of Laboratory Animals of the Ministry of Science and Technology of the People’s Republic of China. Euthanasia was performed under isoflurane-induced anesthesia via parenteral pentobarbitone injection, as previously described (29).

### 2.2 Sample detection

From 2023 to 2024, a total of 408 tissue samples (liver and thymus) were collected from chickens exhibiting clinical symptoms (growth retardation, lethargy, thymic atrophy, pale bone marrow) from six provinces of China: Hunan, Guangdong, Guangxi Zhuang Autonomous Region, Fujian, Yunnan, and Hainan. The detailed distribution of samples across these regions is summarized in Table 1. Tissue samples were homogenized in phosphate-buffered saline (PBS). Suspensions underwent three freeze–thaw cycles, followed by centrifugation at 6000 × *g* for 5 min. Clarified supernatants were collected for DNA extraction. Viral DNA was extracted from the supernatants using the HiPure Viral RNA/DNA Kit (Magen, Guangzhou, China) according to the manufacturer’s instructions. The extracted DNA was screened for CIAV using a real-time fluorescence quantitative polymerase chain reaction (qPCR) assay that targets a conserved region of the vp2 gene. The qPCR was performed using specific primers CAV-qF (5′-ATC AAC CCA AGC CTC CCT-3′), CAV-qR (5′-CTC GTC TTG CCA TCT TAC AG-3′), and CAV-qP probe (5′-FAM-TAC CAC TAC TCC CAG CCG ACC CC-BHQ-3′). Thermal cycling conditions were: initial denaturation at 95 °C for 5 min, denaturation at 95 °C for 15 s, and annealing and extension at 60 °C for 30 s, with 40 cycles (30). qPCR-positive supernatants were filtered using 0.22 μm filter and inoculated onto MDCC-MSB1 monolayers (a lymphoblastoid cell line transformed by Marek’s disease virus) for viral isolation (31).

**Table 1:** Regional distribution of clinical samples collected for CIAV surveillance in Southern China (2023–2024).

Regions	Positive	Total	Positive rate
Guangdong	25	144	17.36%
Hainan	10	10	100.00%
Fujian	26	48	54.17%
Guangxi	68	140	48.57%
Yunnan	13	13	100.00%
Hunan	11	53	20.75%
Total	153	408	37.5%

### 2.3 Immunofluorescence assay (IFA)

MDCC-MSB1 cells were seeded into 96-well plates and infected with CIAV strain WSFL24 at a multiplicity of infection (MOI) of 1, and incubated at 37 °C under 5% CO_2_ for 48 h. Subsequently, the cells were washed twice with PBS and fixed in ice-cold 4% paraformaldehyde at 4 °C for 30 min. Fixed cells were incubated with anti-VP2 monoclonal antibody (kindly provided by the Key Laboratory of Animal Epidemiology of the Ministry of Agriculture and Rural Affairs, China Agricultural University) at 37 °C for 2 h. After washing, cells were incubated with FITC-conjugated anti-mouse IgG secondary antibody (Sigma Aldrich, St. Louis, MO, USA) at 37 °C for 1 h. After washing, cells were visualized using a fluorescence microscope (DMi8, Leica, Germany).

### 2.4 Genome sequencing and phylogenetic analysis

Viral DNA from CIAV-positive samples served as template for PCR amplification. The *vp1* gene was amplified using specific primers: CIAV-VP1F (forward: 5′-ATG GCA AGA CGA GCT CGC AG-3′) and CIAV-VP1R (reverse: 5′-TCA GGG CTG CGA CCC CCA GTA-3′). The complete viral genome was amplified using primers: CAV-F (forward: 5′-GAA TTC GCA TTC CGA GTG GTT ACT ATT CC-3′) and CAV-R (reverse: 5′-GAA TTC GAT TGT GCG ATA AAG CAA TTT GCT-3′) (32, 33). PCR were performed in a 50 μL volume containing 10 pmol of each specific primer, 2 μL of template DNA, and 25 μL of PrimeSTAR HS Premix (TaKaRa). All PCR products were analyzed by 1% agarose gels electrophoresis. Target bands were excised and purified using a gel extraction kit (Solarbio, China). Purified PCR products were subjected to A-tailed using a DNA A-Tailing Kit (TaKaRa). A-tailed products were subsequently ligated into pMD19-T vector (TaKaRa) and transformed into *Escherichia coli* DH5α competent cells. Three colonies were sequenced by Shanghai Sangon Biotech Co., Ltd. (Shanghai, China).

The genomic nucleotide sequences of CIAV isolates were assembled and analyzed using the Seqman program of DNASTAR Lasergene 7.1 software (DNASTAR, Madison, WI, USA). Sequence alignment based on the *vp1*, *vp2*, and *vp3* genes were performed using MEGA11 software. Phylogenetic analysis based on the nucleotide sequences of *vp1* gene and complete genome were performed between isolates and 22 reference strains (Table 2) using the neighbor-joining method with 1,000 bootstrap replicates (34).

**Table 2:** CIAV reference strains retrieved from the GenBank database.

CIAV strain	Accession No.	Country	Genotype	Year
CAU269-7	AF227982	Australia	I	2001
3,711	EF683159	Australia	I	2007
LF4	AY839944	China	II	2005
HLJ15108	KY486137	China	II	2018
AH4	DQ124936	China	II	2005
CAV-EG-14	MH001565	Egypt	II	2018
SD1403	KU221054	China	II	2016
SD15	KX811526.1	China	II	2017
GD-103	KU050678	China	IIIa	2016
GD-102	KU050677	China	IIIa	2016
HLJ14101	KY486136	China	IIIa	2018
JL14023	KY486145	China	IIIa	2018
JS15165	KY486152	China	IIIa	2018
SC-HY	KM496303	China	IIIa	2014
SD1515	KU645515	China	IIIa	2016
Cux-1	M55918	Germany	IIIb	2008
26P4	D10068	The Netherlands	IIIb	2007
SD22	DQ141673	China	IV	2005
SD24	AY999018	China	IV	2005
HLJ15170	KY4861144	China	V	2018
JS211949	PP355003	China	V	2024
CQ21313	OP038237	China	V	2023
HNHYL23	PQ610960	Isolated in this study	IIIa	2023
HNHCY23	PQ610959	Isolated in this study	IIIa	2023
HNZZZ23	PQ610991	Isolated in this study	IIIa	2023
FJLQQ23	PQ610925	Isolated in this study	IIIa	2023
FJCLY23	PQ610924	Isolated in this study	IIIa	2024
FJCHF23	PQ610923	Isolated in this study	IIIa	2023
HNZHH23	PQ610988	Isolated in this study	IIIa	2023
HNDHJ23	PQ610993	Isolated in this study	IIIa	2023
GXSYC23	PQ610954	Isolated in this study	IIIa	2023
GXMQA23	PQ610957	Isolated in this study	IIIa	2023
GDDZX23	PQ610944	Isolated in this study	IIIa	2023
GDHWH23	PQ610946	Isolated in this study	IIIa	2023
GDHAS23	PQ610945	Isolated in this study	IIIa	2023
FJHYLRJ23	PQ610926	Isolated in this study	V	2023
HNLLX24	PQ610961	Isolated in this study	V	2024
HNCFF23	PQ610958	Isolated in this study	V	2023
WSFL24	PQ610992	Isolated in this study	V	2024
CZYHZ24	PQ610922	Isolated in this study	V	2024
GDGZY23	PQ610941	Isolated in this study	V	2023
GDLQQ23	PQ610943	Isolated in this study	V	2023
GDLCZ23	PQ610942	Isolated in this study	V	2023

### 2.5 Animal experiment design

To assess the pathogenicity of the isolate in this study, a total of ninty 1-day-old Specific Pathogen-Free (SPF) chicks were randomly divided into three groups (group I, II, and III, 30 chicks per group). Chicks in group I and II were inoculated with Cux-1 strain (genotype IIIb, 10^5^ EID_50_ per chick) and WSFL-24 strain (genotype V, 10^5^ EID_50_ per chick), respectively. While chicks in group III received an equal volume of PBS as controls. To accurately assess survival rates, a subset of 10 marked chicks per group was reserved solely for mortality observation and was not subjected to scheduled euthanasia. The remaining birds were used for pathological and virological analyses. All clinical monitoring, sample collection, and evaluations were performed by investigators blinded to group assignments. The birds in each group were maintained in independently negative pressurized isolators. Food and water were provided ad libitum. Clinical manifestations of this disease and mortality were monitored daily. At 12, 14, and 18 days post-inoculation (dpi), three chickens in each group were euthanized for necropsy. Before euthanasia, chickens were rendered unconscious and anesthesia was maintained using isoflurane delivered in oxygen. Euthanasia was then performed by intravenous or intraperitoneal injection of pentobarbitone sodium (100 mg/kg) as previously described (29). Thymus, bursa of Fabricius, bone marrow, and anticoagulated blood samples were collected to assess the thymus index, bursa of Fabricius index, and hematocrit. Thymus index was calculated as follows: Thymus index (%) = [thymus weight (g) / body weight (g)] × 100. Bursa index was calculated as follows: Bursa index (%) = [bursa of fabricius weight (g) / body weight (g)] × 100. Hematocrit (HCT) was measured in EDTA-anticoagulated blood. HCT < 27.0% defined anemia. To further confirm the pathogenicity of the isolate, thymus tissues were fixed in 10% neutral-buffered formalin for 24 h. Tissues were dehydrated through graded ethanol and xylene, embedded in paraffin wax, sectioned at 5 μm thickness, and stained with hematoxylin and eosin (H&E) for histopathological evaluation under a light microscope. For viral tissue distribution analysis, thymus and bursa tissues were homogenized in PBS, and the homogenates were centrifuged to obtain the supernatant. Supernatant DNA was extracted from the supernatant. The extracted DNA was analyzed by qPCR, and viral load was calculated.

### 2.6 Data analysis

The data are presented as mean ± standard deviation (SD). Statistical analyses were performed using GraphPad Prism software (version 8.0). A two-way analysis of variance (ANOVA) was employed to evaluate the effects of two independent factors—treatment group (Mock, Cux-1, and WSFL24) and time post-inoculation (12, 14, and 18 dpi)—on continuous variables, including hematocrit values, organ indices, and viral loads. The model also included an interaction term between treatment group and time to assess potential combined effects. When significant differences were detected, *p*-values < 0.05 were considered statistically significant and are denoted by asterisks (*) in the figures.

## 3 Results

### 3.1 Virus detection, isolation, and identification

**Figure 1:**
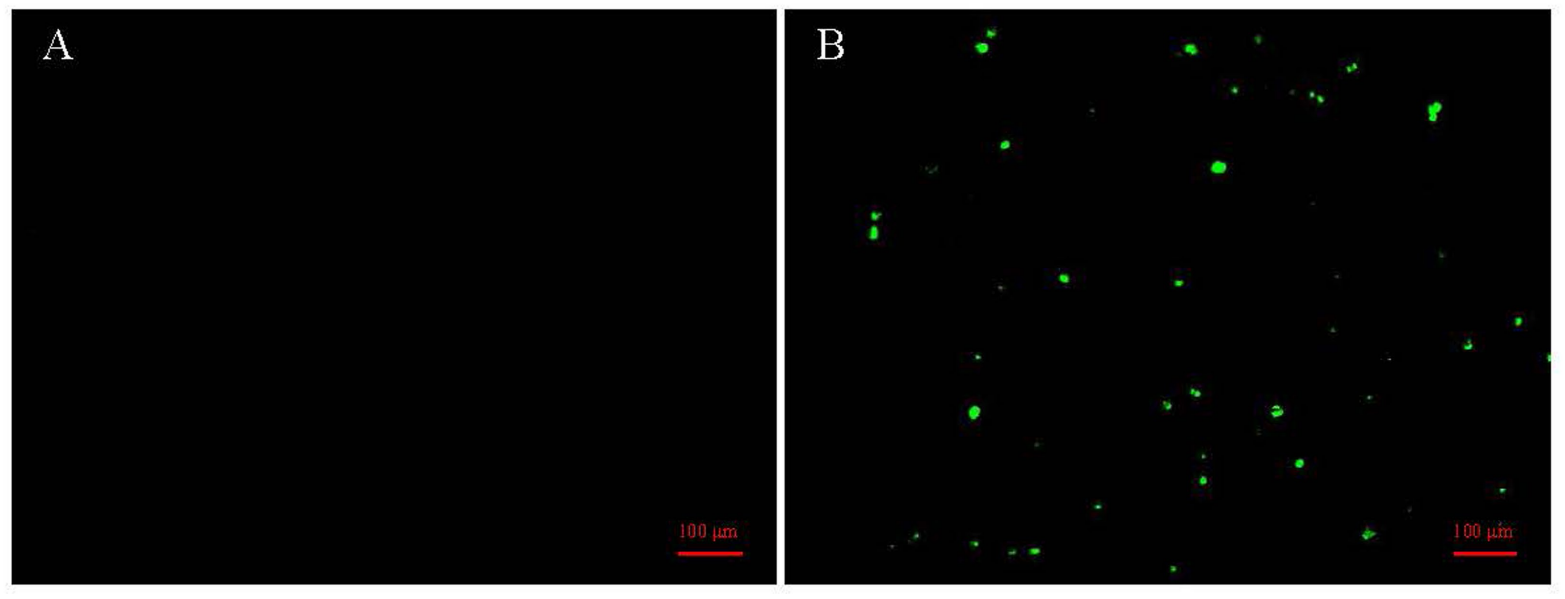
Detection of CIAV in MDCC-MSB1 cells by immunofluorescence assay (IFA). **(A)** Negative control MDCC-MSB1 cells inoculated with PBS, showing no specific fluorescence signals. **(B)** MDCC-MSB1 cells inoculated with the WSFL24 strain (MOI = 1) for 48 h, showing distinct cytoplasmic green fluorescence indicative of viral infection. Cells were stained with an anti-VP2 monoclonal antibody and detected with a FITC-conjugated secondary antibody.

From 2023 to 2024, a total of 408 clinical samples were collected from chickens exhibiting suspected CIAV infection in southern China. These samples were screened for CIAV using qPCR assay. As a result, 153 samples tested positive for CIAV, indicating a 37.5% positivity rate. Regional prevalence varied considerably, ranging from 17.36% (Guangdong) to 100% (Hainan and Yunnan), as detailed in Table 1. Virus isolation was performed through three serial blind passages using MDCC-MSB1 cells, and identified using IFA assay. As a result, specific cytoplasmic green fluorescence signals were observed in CIAV-infected cells (Figure 1). These findings demonstrated a considerable prevalence of CIAV in poultry industry in southern China and underscore the ongoing circulation and potential threat of CIAV within the Chinese poultry industry.

### 3.2 Genome characterization and phylogenetic analysis

**Figure 2:**
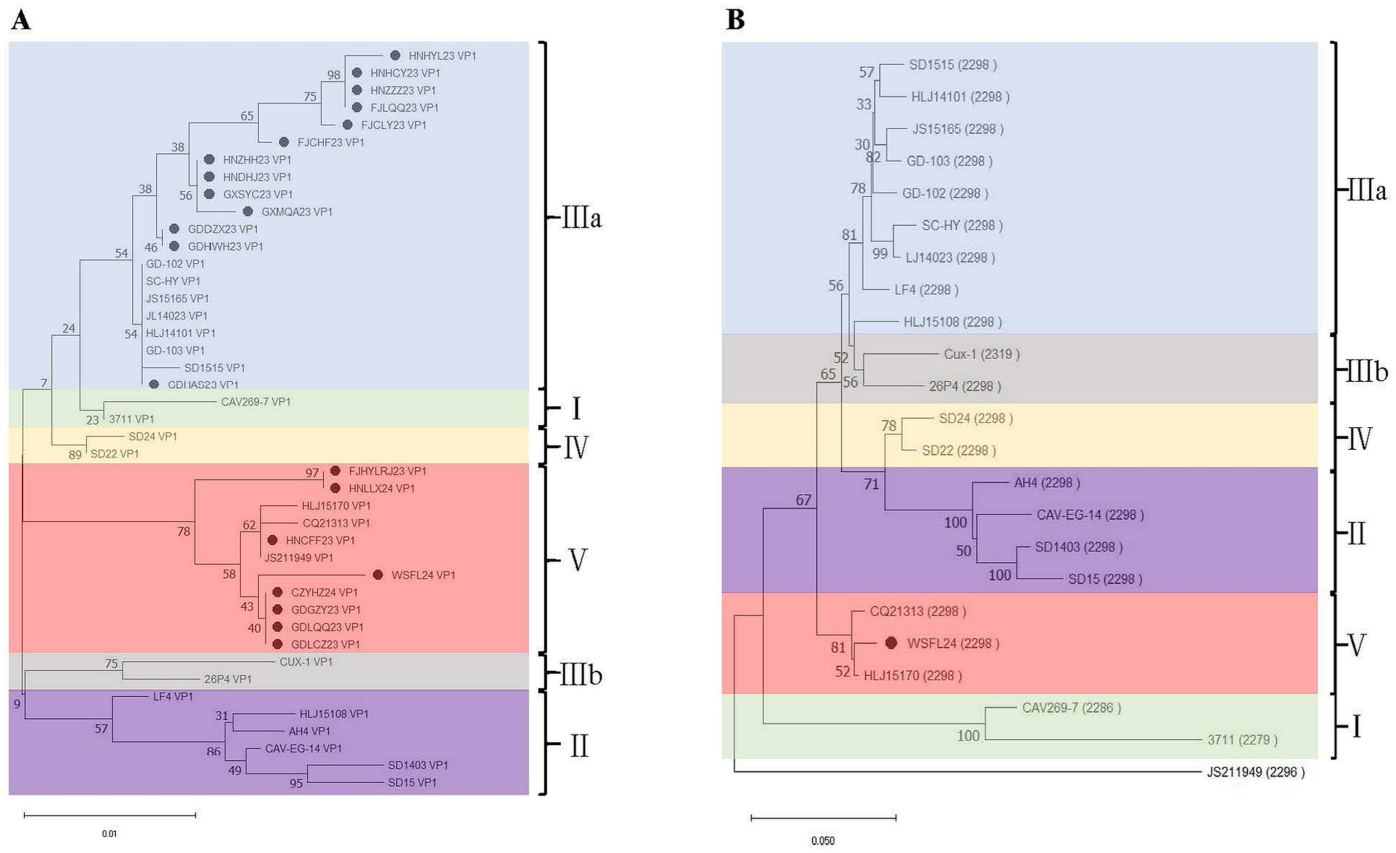
Phylogenetic analysis of CIAV isolates from southern China (2023–2024). **(A)** Phylogenetic tree based on complete vp1 gene nucleotide sequences. **(B)** Genome-wide phylogenetic analysis of the representative WSFL24 strain (genotype V) compared with reference strains from different genotypes. Isolates obtained in this study are marked with solid circles.

Phylogenetic analysis of vp1 gene sequences of 21 CIAV isolates and 22 reference strains was performed using MEGA software. As a results, 13 isolates (HNHYL23, HNHCY23, HNZZZ23, FJLQQ23, FJCLY23, HNZHH23, HNDHJ23, GXSYC23, GXMQA23, GDDZX23, GDHWH23, and GDHAS23) belonged to genotype IIIa, and other eight isolates (FJHYLRJ23, HNLLX24, HNCFF23, WSFL24, CZYHZ24, GDGZY23, GDLQQ23, and GDLCZ23) were classified into genotype V (Figure 2). During clinical sampling and isolation, the WSFL24 strain demonstrated strong pathogenicity and enhanced cellular adaptability compared to other isolates. Furthermore, as a member of the less frequently reported genotype V, this strain was selected for further characterization.

To characterize the amino acid of VP1, VP2, and VP3 of WSFL24 isolate, a multiple sequence alignment based on the amino acid sequence of VP1, VP2, and VP3 between WSFL24 and 22 reference strains was conducted using the Clustal X program. A multiple sequence alignment revealed 31, 9, and 17 amino acid substitutions in the VP1, VP2, and VP3 proteins of WSFL24, respectively, compared to the 22 reference strains (Supplementary Tables S1–S3). The residue 370 in VP1 was the most hypervariable site, exhibiting five variants: alanine (A), glycine (G), threonine (T), serine (S), and arginine (R) (Supplementary Table S1). Notably, the amino acid residues H294, A370, and I436 were conserved in genotype V strains (Supplementary Table S1).

### 3.3 Pathogenicity analysis of WSFL24

**Figure 3:**
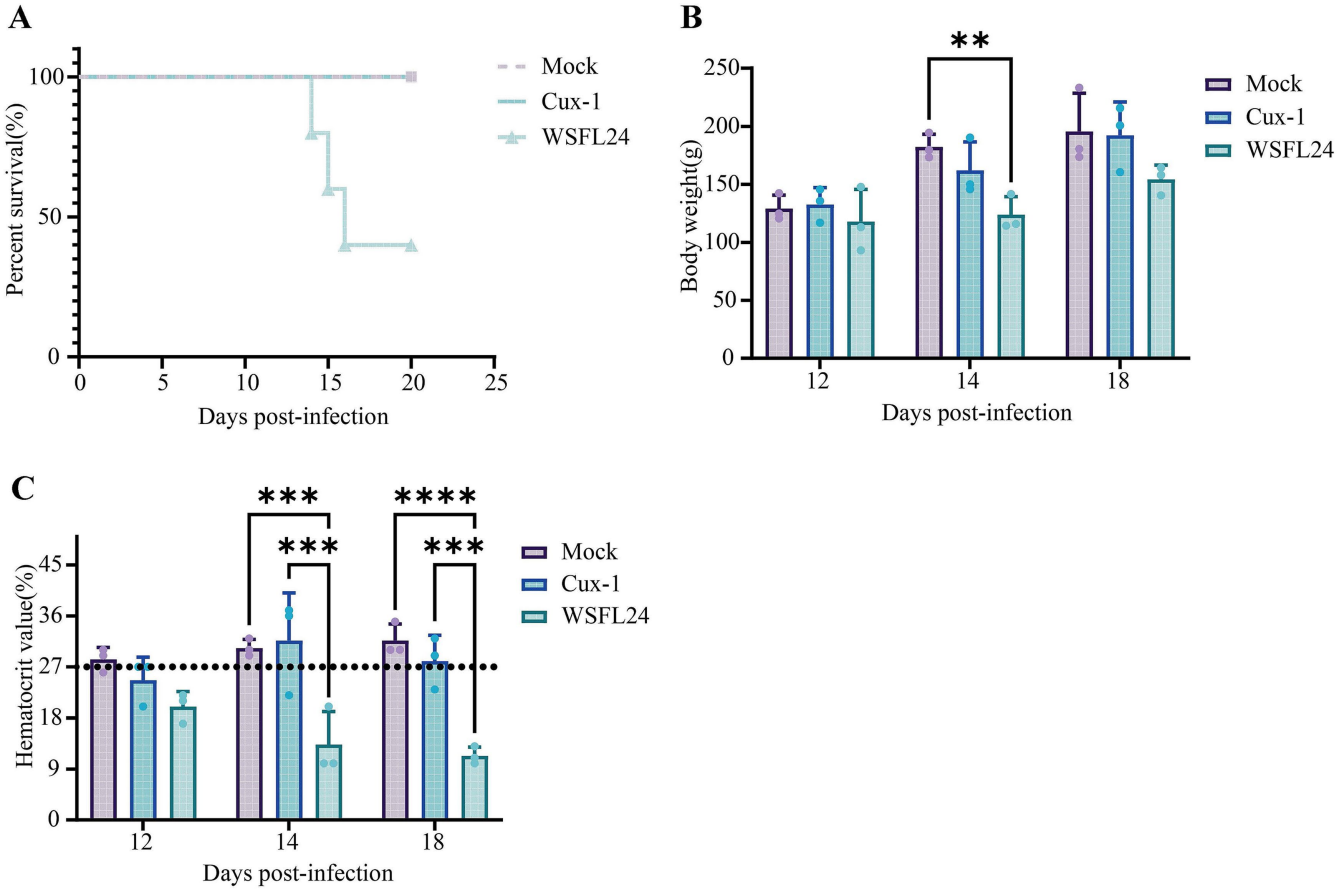
Pathogenicity assessment of the WSFL24 strain in SPF chicks. **(A)** Survival rates of chicks inoculated with WSFL24, Cux-1, or PBS (Mock) (*n* = 10 per group). **(B)** Body weight changes of chicks at 12, 14, and 18 dpi (*n* = 3 per group per time point). **(C)** Hematocrit (HCT) levels of chicks during the experimental period (*n* = 3 per group per time point). Data are expressed as means ± SD.

To evaluate the pathogenicity of the WSFL24 strain, animal experiments were performed using SPF chickens. As a result, compared to the mock-infected chickens, the Cux-1-infected chickens exhibited mild depression and reduced activity but no mortality throughout the study. In contrast, WSFL24-infected chickens showed reduced body weight, succumbed to infection beginning at 14 dpi, and reached 60% mortality between 14 and 16 dpi (Figures 3A,B). Using the established anemia threshold [hematocrit (HCT) < 27%] (35), WSFL24-infected chickens displayed HCT values below 27% at both 14 and 18 dpi, confirming severe anemia in this group (Figure 3C). No significant hematocrit reduction was observed in control or Cux-1-infected chickens.

**Figure 4:**
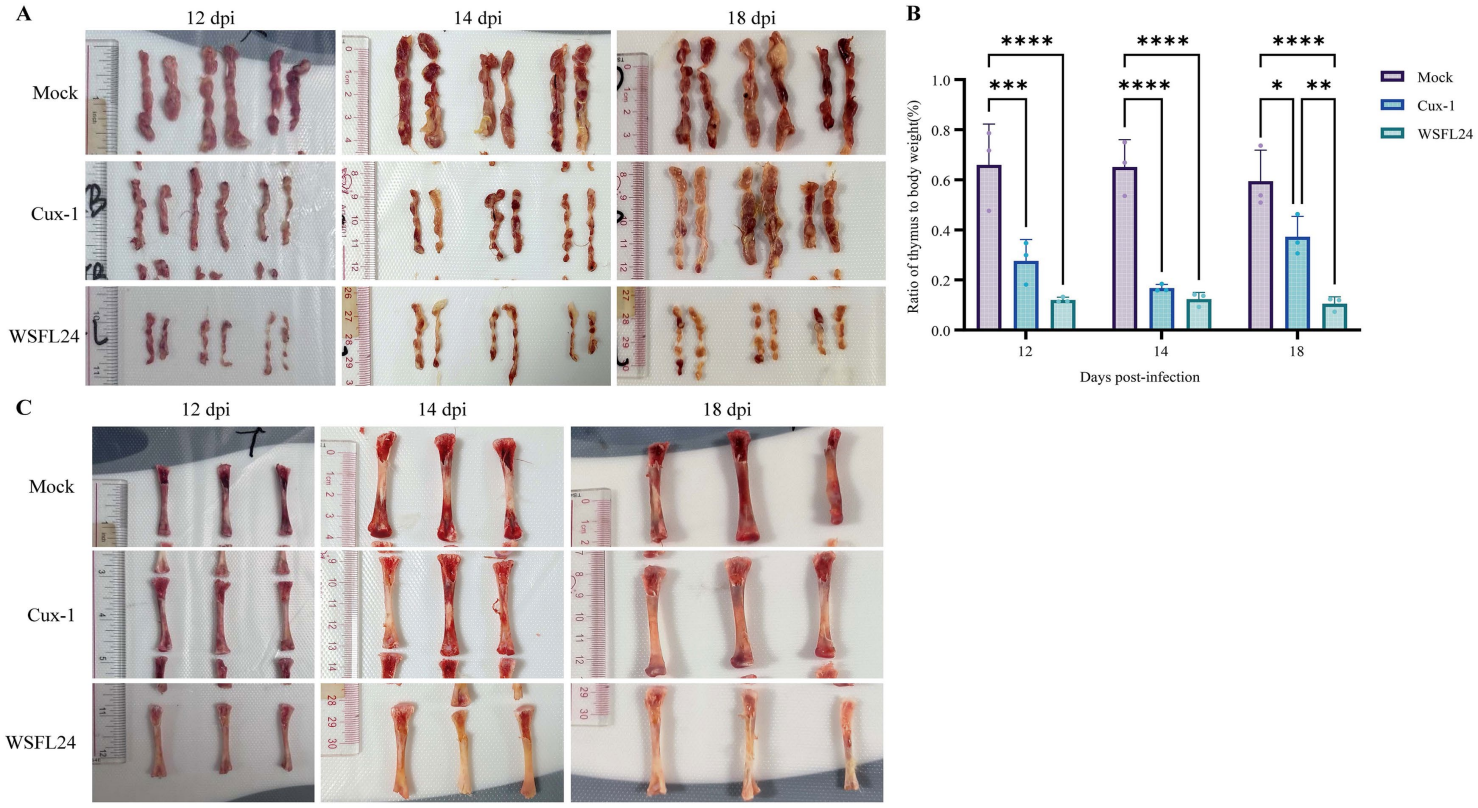
Gross pathological lesions observed in CIAV-infected chicks. **(A)** Representative gross morphology of the thymus in mock-infected and infected chicks at 12, 14, and 18 dpi. **(B)** Thymus indices of chicks in each group (*n* = 3 per group per time point). Data are expressed as means ± SD. **(C)** Representative gross morphology of the bone marrow in mock-infected and infected chicks at 12, 14, and 18 dpi.

At necropsy, both Cux-1- and WSFL24-infected chickens exhibited thymic atrophy compared to controls (Figure 4A). Furthermore, the thymus index of WSFL24-infected chickens was significantly lower than that of Cux-1-infected group at 18 dpi (Figure 4B), indicating more pronounced thymic atrophy in WSFL24-infected chickens. Considerring the observed clicical signs of anemia and reduced HCT, we performed gross examination of bone marrow at 12, 14, and 18 dpi. Notably, WSFL24-infected chickens exhibited progressive bone marrow yellowing compared to controls (Figure 4C), consistent with impaired erythropoiesis and advanced anemia. These findings collectively demonstrate the heightened virulence of the WSFL24 strain.

**Figure 5:**
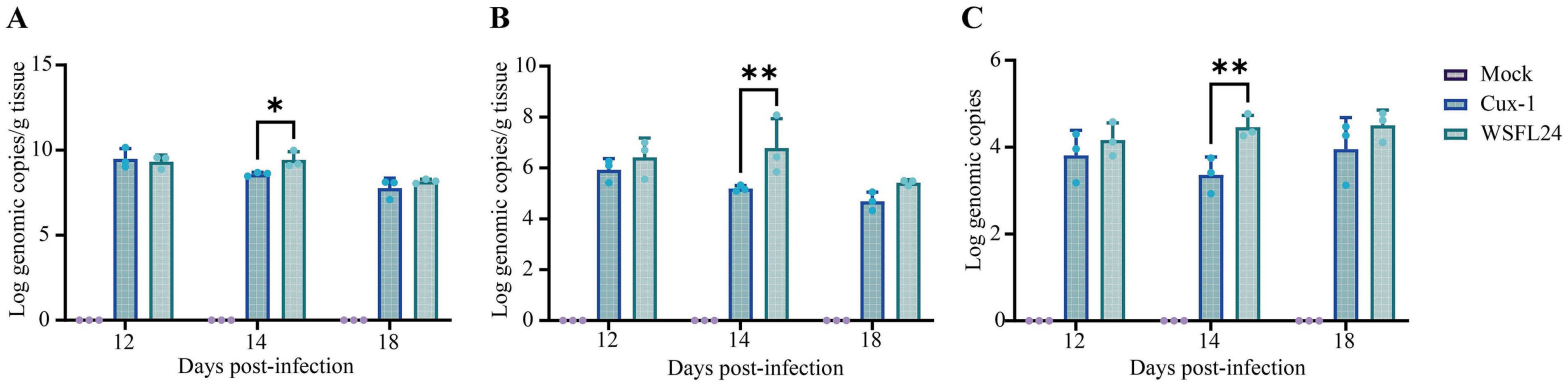
Quantification of viral loads in tissues and cloacal swabs of CIAV-infected chicks. **(A)** Viral loads in thymus tissues (log_10_ copies/g DNA). **(B)** Viral loads in bursa of Fabricius tissues (log_10_ copies/g DNA). **(C)** Viral shedding dynamics in cloacal swabs (log_10_ copies DNA). Data are expressed as means ± SD (*n* = 3 per group per time point).

To further assess viral replication kinetics and tissue distribution, quantitative viral loads were determined in the thymus, bursa of Fabricius, and cloacal swabs at 12, 14, and 18 days post-infection (dpi). At 14 dpi, viral loads in both thymus and bursal tissues were markedly higher in the WSFL24-infected group than in the Cux-1-infected group. Specifically, thymic viral loads reached 9.42 ± 0.49 log_10_ copies/g in WSFL24-infected chickens, compared with 8.59 ± 0.11 log_10_ copies/g in Cux-1-infected chickens (*p* < 0.01) (Figure 5A). Similarly, bursal viral loads were significantly higher in the WSFL24 group (6.78 ± 1.16 log_10_ copies/g) than in the Cux-1 group (5.19 ± 0.12 log_10_ copies/g, *p* < 0.01) (Figure 5B). Consistently, viral shedding detected in cloacal swabs was also significantly greater in the WSFL24 group (4.46 ± 0.27 log_10_ copies) compared with the Cux-1 group (3.36 ± 0.41 log_10_ copies, *p* < 0.05) at 14 dpi (Figure 5C). Collectively, these quantitative results demonstrate that the WSFL24 strain exhibits enhanced replication capacity and broader tissue dissemination compared with the classical Cux-1 strain.

**Figure 6:**
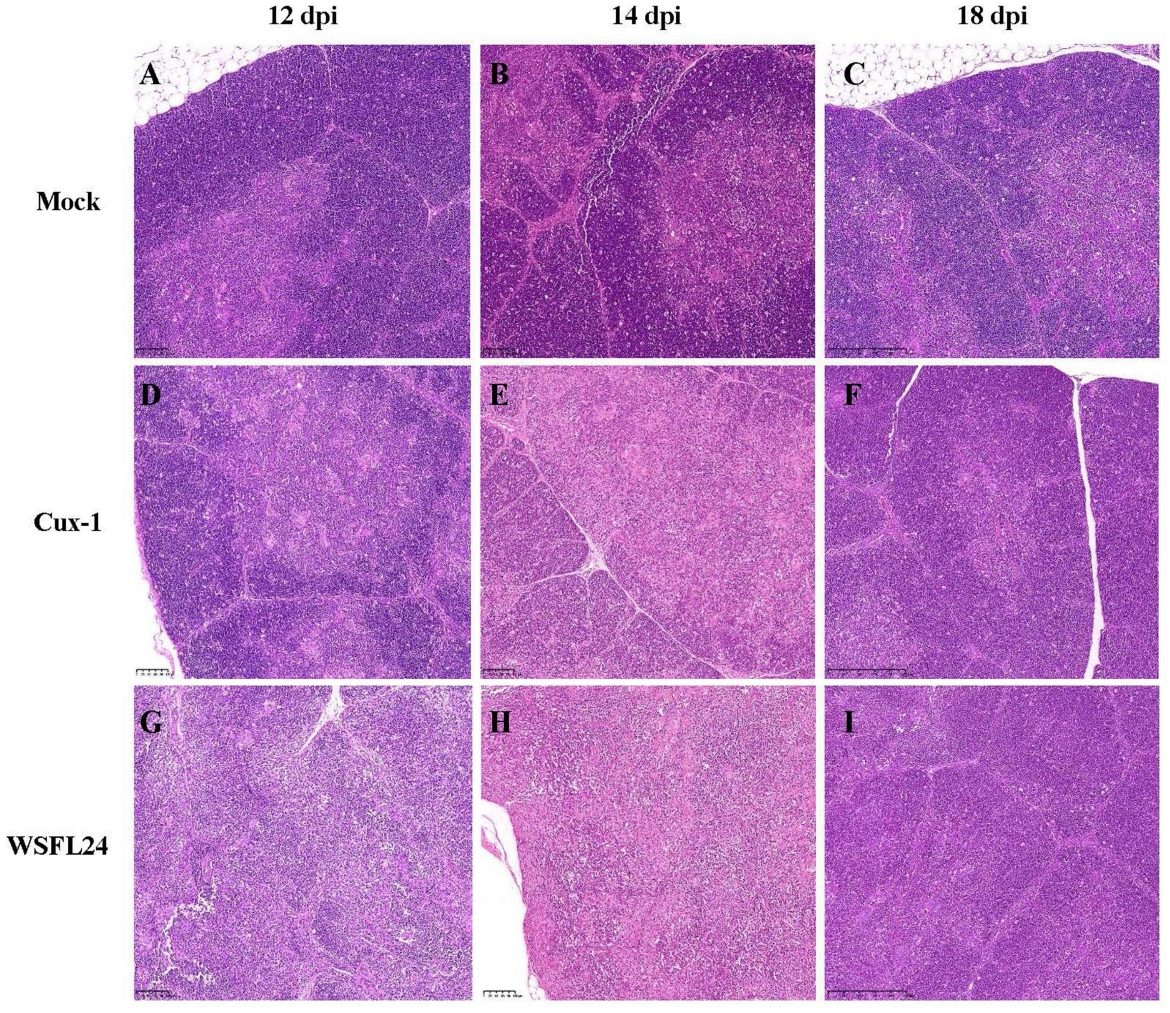
Histopathological changes in thymus tissues of CIAV-infected chicks. **(A–C)** Mock-infected group at 12, 14, and 18 dpi, showing normal corticomedullary structure. **(D–F)** Cux-1-infected group at 12, 14, and 18 dpi, showing mild cortical disorganization. **(G–I)** WSFL24-infected group at 12, 14, and 18 dpi, showing severe cortical disorganization and loss of corticomedullary junction integrity.

To further evaluate the pathogenicity of the WSFL24 strain in SPF chickens, histopathological analysis of thymic tissue was performed. Consistent with gross necropsy observations, histological examination revealed distinct temporal and group-specific pathological changes (Figure 6). At 12 dpi, thymic sections from WSFL24-infected chickens exhibited disorganized cortical architecture and loss of corticomedullary junction definition (Figure 6H), whereas no abnormalities were observed in mock-infected group (Figure 6A) or Cux-1-infected group (Figure 6D). By 14 dpi, both WSFL24-infected (Figure 6I) and Cux-1-infected (Figure 6E) chickens displayed cortical disorganization and indistinct corticomedullary junctions compared to controls (Figure 6B). At 18 dpi, only mild blurring of the corticomedullary junction persisted in WSFL24-infected chickens (Figure 6J), indicating partial histological recovery despite prior severe atrophy.

## 4 Discussion

Chicken infectious anemia (CIA), caused by chicken infectious anemia virus (CIAV), is an immunosuppressive disease with globally distribution, which significantly affects poultry health and production (36). Since its initial discovery in Japan in 1979, CIAV has been reported worldwide, causing substantial impacts on the poultry industry (7, 9). CIAV was first reported in China in 1996. Subsequently, CIAV-positive cases have been documented across all provinces of China, resulting in substantial economic losses (6, 32, 33, 37).

In the present study, we conducted an epidemiological survey of CIAV in southern China from 2023 to 2024. Molecular detection and sequencing revealed a relatively high positivity rate of 37.5%. Subsequent phylogenetic analysis based on VP1 amino acid sequences from 21 field isolates, along with 22 reference strains, demonstrated that 13 isolates clustered into genotype IIIa, while 8 isolates grouped within genotype V. Compared to previous epidemiological reports, which identified genotype IIIa as the dominant lineage in China (37–39), our findings reveal a significant presence of genotype V strains in southern China. This is consistent with recent reports from Jiangsu Province, where Zhang et al. (47) documented genotype V infections between 2020 and 2022. Collectively, these observations suggest the ongoing geographical expansion and increased detection of genotype V in certain regions of China.

To further explore its biological characteristics, we selected the genotype V strain WSFL24 for sequence comparison and pathogenicity evaluation. Among the three proteins encoded by CIAV, VP1 is the sole structural component and exhibits the highest sequence variability, influencing both viral replication and pathogenicity. In this study, VP1 of WSFL24 displayed 31 amino acid substitutions compared to reference strains. Notably, it retained glutamine (Q) at residues 139 and 144 previously implicated in efficient viral replication (22). Notably, Yamaguchi et al. (40) identified VP1 residue 394 as a virulence determinant, with Q394 correlating with high pathogenicity. WSFL24 also possesses Q at this position, indicating high virulence potential.

To validate this hypothesis, pathogenicity experiments were performed using one-day-old SPF chicks. Infection with the WSFL24 strain resulted in a 60% mortality rate, which was markedly higher than that observed in the reference Cux-1 strain group, in which no deaths occurred. In addition, WSFL24 infection caused more severe anemia, greater viral loads in lymphoid tissues and the cloaca, and more pronounced thymic atrophy compared with Cux-1 infection. CAV antigen was initially detected in the thymus and bone marrow between 4 and 7 days post-inoculation (dpi) in WSFL24-infected chicks, indicating early viral replication in hematopoietic and immune organs. This observation is consistent with the known biology of chicken anemia virus (CAV), which—like many other DNA viruses—interferes with or evades host antiviral pathways and exploits host cellular machinery for the synthesis of viral gene products (41). These findings are consistent with its molecular features and support its classification as a highly virulent strain In contrast, although the Cux-1 strain is widely used as a reference in pathogenicity comparisons, it exhibits consistently attenuated virulence than Chinese field isolates (32, 42–44). Overall, these results provide fundamental insights into the molecular evolution and pathogenic mechanisms of CIAV, emphasizing the necessity of ongoing molecular surveillance and the development of updated vaccine candidates to address emerging virulent genotypes.

Our findings align with Song et al. (48) reporting the highly virulent 17AD008 strain, causing severe anemia and mortality in SPF chicks. While Chinese isolates generally exhibit higher pathogenicity than strains from Southeast Asia, mortality rates exceeding 60%—as observed for WSFL24—remain uncommon. For example, even under high-dose challenge conditions, the SDTY2021-TJ strain induced only 50% mortality (45). Notably, Fang et al. (46) observed elevated virulence of SD15 strain, further supporting the notion that highly pathogenic CIAV strains are emerging in China.

In summary, our study provides new insights into the molecular epidemiology of CIAV circulating in southern China. The increased prevalence of genotype V strains and identification of the highly virulent WSFL24 isolate suggest an ongoing shift in the regional genetic landscape. These findings offer foundational insights into CIAV’s molecular evolution and pathogenic mechanisms, while emphasizing the urgent need for continued molecular surveillance to monitor the dissemination of genotype V strains. Moreover, our findings indicate that current vaccine strains may offer suboptimal protection against emerging highly virulent genotypes, highlighting the necessity for updated and regionally matched vaccine formulations.

## Data Availability

The datasets presented in this study can be found in online repositories. The names of the repository/repositories and accession number(s) can be found in the article/Supplementary material.
