# Supplementary material for: From surveillance to pathogenesis: characterization of genotype V of chicken infectious anemia virus
Source: Front Vet Sci. 2025 Nov 18;12:1710392. doi: 10.3389/fvets.2025.1710392 (PMC12670581; doi:10.3389/fvets.2025.1710392)
Supplement: Supplementary file 1 [file Table_1.docx]

**Supplementary Table S1. Amino acid sequence comparison of VP1 protein.**

| CIAV Strain | Genotype | Amino acid positions | | | | | | | | | | | | | | | | | | | | | | | | | | | | | | |
| --- | --- | --- | --- | --- | --- | --- | --- | --- | --- | --- | --- | --- | --- | --- | --- | --- | --- | --- | --- | --- | --- | --- | --- | --- | --- | --- | --- | --- | --- | --- | --- | --- |
|  |  | 14 | 22 | 75 | 97 | 125 | 139 | 144 | 157 | 183 | 210 | 248 | 251 | 254 | 257 | 270 | 287 | 290 | 292 | 294 | 370 | 375 | 376 | 379 | 413 | 423 | 424 | 436 | 444 | 446 | 447 | 448 |
| WSFL24 | Ⅴ | A | H | V | L | L | Q | Q | M | N | F | N | R | E | M | P | T | P | G | H | A | E | L | N | A | C | E | I | C | G | W | Q |
| JS211949 | Ⅴ | A | H | V | L | L | K | E | M | N | F | N | R | E | M | P | T | P | G | H | A | E | L | N | A | W | Q | I | Y | G | S | Q |
| HLJ15170 | Ⅴ | A | Y | V | L | L | K | E | M | N | F | N | R | E | M | P | T | P | G | H | A | E | L | N | A | W | Q | I | Y | G | S | Q |
| CQ21313 | Ⅴ | A | H | V | L | L | K | E | M | N | F | N | R | E | M | P | T | P | G | H | A | E | L | H | A | W | Q | I | Y | G | S | Q |
| SD24 | Ⅳ | A | H | V | M | L | K | E | V | Y | F | N | R | E | M | P | S | A | G | Q | S | E | L | N | A | W | Q | V | Y | G | S | Q |
| SD22 | Ⅳ | A | H | V | M | L | K | E | V | N | F | N | R | E | M | P | S | A | G | Q | S | E | L | N | A | W | Q | V | Y | G | S | Q |
| SD1515 | Ⅲa | A | H | V | M | L | K | E | V | N | F | N | R | E | M | P | S | A | A | Q | G | E | I | N | S | W | Q | V | Y | G | S | Q |
| SC-HY | Ⅲa | A | H | V | M | L | K | E | V | N | F | N | R | E | M | P | S | A | G | Q | G | E | I | N | S | W | Q | V | Y | G | S | Q |
| JS15165 | Ⅲa | A | H | V | M | L | K | E | V | N | F | N | R | E | M | P | S | A | G | Q | G | E | I | N | S | W | Q | V | Y | G | S | Q |
| JL14023 | Ⅲa | A | H | V | M | L | K | E | V | N | F | N | R | E | M | P | S | A | G | Q | G | E | I | N | S | W | Q | V | Y | G | S | Q |
| HLJ14101 | Ⅲa | A | H | V | M | L | K | E | V | N | F | N | R | E | M | P | S | A | G | Q | G | E | I | N | S | W | Q | V | Y | G | S | Q |
| GD-103 | Ⅲa | A | H | V | M | L | K | E | V | N | F | N | R | E | M | P | S | A | G | Q | G | E | I | N | S | W | Q | V | Y | G | S | Q |
| GD-102 | Ⅲa | A | H | V | M | L | K | E | V | N | F | N | R | E | M | P | S | A | G | Q | G | E | I | N | S | W | Q | V | Y | G | S | Q |
| Cux-1 | Ⅲb | S | H | V | M | I | K | D | V | N | F | N | Q | G | M | P | A | A | G | Q | S | E | L | N | A | W | Q | V | Y | G | T | Q |
| 26P4 | Ⅲb | A | H | V | M | I | K | E | M | N | F | N | R | G | M | P | T | A | G | Q | S | E | L | N | A | W | Q | V | Y | E | T | Q |
| SD1403 | Ⅱ | A | Q | I | L | I | K | E | V | N | F | N | R | E | T | P | T | P | G | Q | T | K | L | N | A | W | Q | V | Y | G | S | Q |
| SD15 | Ⅱ | A | Q | I | L | I | Q | Q | V | N | S | N | R | E | M | P | T | P | G | Q | T | E | L | N | A | W | Q | V | Y | G | S | P |
| LF4 | Ⅱ | A | H | I | M | L | Q | Q | V | N | F | N | R | E | M | P | S | P | G | Q | S | E | L | N | S | W | Q | V | Y | G | S | Q |
| HLJ15108 | Ⅱ | A | N | I | L | I | Q | Q | V | N | F | N | R | E | M | P | A | A | G | Q | S | E | L | N | A | W | Q | V | Y | G | S | Q |
| AH4 | Ⅱ | A | H | I | L | I | Q | Q | V | N | F | S | R | E | M | P | T | A | G | Q | S | E | L | N | A | W | Q | V | Y | G | S | Q |
| CAV-EG-14 | Ⅱ | A | H | I | L | I | Q | Q | V | N | F | N | R | E | M | P | T | P | G | Q | S | E | L | N | A | W | Q | V | Y | G | S | Q |
| CAU269-7 | Ⅰ | A | H | V | M | I | K | E | V | N | F | N | R | E | M | L | T | A | G | Q | R | E | L | N | S | W | Q | V | Y | G | S | Q |
| 3711 | Ⅰ | A | H | V | M | I | K | E | V | N | F | N | R | E | M | P | S | A | G | Q | G | E | L | N | S | W | Q | V | Y | G | S | Q |
