# Supplementary material for: From surveillance to pathogenesis: characterization of genotype V of chicken infectious anemia virus
Source: Front Vet Sci. 2025 Nov 18;12:1710392. doi: 10.3389/fvets.2025.1710392 (PMC12670581; doi:10.3389/fvets.2025.1710392)
Supplement: Supplementary file 2 [file Table_2.docx]

**Supplementary Table S2. Amino acid sequence comparison of VP2 protein.**

| Strain | Genotype | Amino acid positions | | | | | | | | |
| --- | --- | --- | --- | --- | --- | --- | --- | --- | --- | --- |
|  |  | 14 | 43 | 77 | 126 | 153 | 155 | 177 | 180 | 186 |
| WSFL24 | Ⅴ | E | Y | N | R | V | K | G | T | E |
| JS211949 | Ⅴ | E | Y | N | R | V | K | G | T | E |
| HLJ15170 | Ⅴ | E | Y | N | R | V | K | G | T | E |
| CQ21313 | Ⅴ | E | Y | N | R | V | R | G | T | E |
| SD24 | Ⅳ | E | Y | N | R | V | K | G | T | E |
| SD22 | Ⅳ | E | Y | N | R | V | K | G | T | E |
| SD1515 | Ⅲa | K | Y | N | R | V | K | G | T | E |
| SC-HY | Ⅲa | E | Y | N | R | V | K | G | T | E |
| JS15165 | Ⅲa | E | Y | N | R | V | K | G | T | E |
| JL14023 | Ⅲa | E | Y | N | R | V | K | G | T | E |
| HLJ14101 | Ⅲa | E | Y | N | R | V | K | G | T | E |
| GD-103 | Ⅲa | E | Y | N | R | V | K | G | T | E |
| GD-102 | Ⅲa | E | Y | N | R | V | K | G | T | E |
| Cux-1 | Ⅲb | E | Y | N | R | A | K | G | T | E |
| 26P4 | Ⅲb | E | Y | N | R | V | K | G | T | G |
| SD1403 | Ⅱ | E | F | N | R | V | K | A | S | E |
| SD15 | Ⅱ | E | Y | N | R | V | K | A | S | E |
| LF4 | Ⅱ | E | Y | N | R | V | K | G | T | E |
| HLJ15108 | Ⅱ | E | Y | N | R | V | K | G | T | E |
| AH4 | Ⅱ | E | Y | N | R | V | K | G | T | E |
| CAV-EG-14 | Ⅱ | E | Y | N | R | V | K | G | T | E |
| CAU269-7 | Ⅰ | E | Y | S | R | V | K | G | T | E |
| 3711 | Ⅰ | E | Y | N | L | V | K | G | T | E |
