# Supplementary material for: From surveillance to pathogenesis: characterization of genotype V of chicken infectious anemia virus
Source: Front Vet Sci. 2025 Nov 18;12:1710392. doi: 10.3389/fvets.2025.1710392 (PMC12670581; doi:10.3389/fvets.2025.1710392)
Supplement: Supplementary file 3 [file Table_3.docx]

**Supplementary Table S3. Amino acid sequence comparison of VP3 protein.**

| CIAV Strain | Genotype | Amino acid positions | | | | | | | | | | | | | | | | | |
| --- | --- | --- | --- | --- | --- | --- | --- | --- | --- | --- | --- | --- | --- | --- | --- | --- | --- | --- | --- |
|  |  | 2 | 4 | 6 | 8 | 25 | 42 | 53 | 54 | 64 | 67 | 70 | 73 | 91 | 103 | 116 | 118 | | 120 |
| WSFL24 | Ⅴ | N | L | E | T | L | I | R | D | N | S | F | V | D | S | R | C | | R |
| JS211949 | Ⅴ | N | L | E | T | L | I | R | A | N | S | F | V | D | S | R | C | | R |
| HLJ15170 | Ⅴ | N | L | E | T | L | I | R | A | N | S | F | V | D | S | R | C | | R |
| CQ21313 | Ⅴ | N | L | E | T | L | I | R | A | N | S | F | V | D | S | R | C | | G |
| SD24 | Ⅳ | N | L | E | T | L | I | R | A | N | S | F | V | D | S | R | C | | R |
| SD22 | Ⅳ | N | L | E | T | L | I | R | A | N | S | F | V | D | S | R | C | | R |
| SD1515 | Ⅲa | N | L | D | T | L | I | R | A | N | S | F | V | D | S | R | C | | R |
| SC-HY | Ⅲa | N | L | E | T | L | I | H | A | N | S | F | V | D | S | R | C | | R |
| JS15165 | Ⅲa | N | L | E | T | L | I | R | A | N | S | F | V | D | S | R | C | | R |
| JL14023 | Ⅲa | N | L | E | T | L | I | R | A | N | S | F | V | D | S | R | C | | R |
| HLJ14101 | Ⅲa | N | L | E | T | L | I | R | A | N | S | F | V | D | S | R | C | | R |
| GD-103 | Ⅲa | N | L | E | T | L | I | R | A | N | S | F | V | D | S | R | C | | R |
| GD-102 | Ⅲa | N | L | E | T | L | I | R | A | N | S | F | V | D | S | R | C | | R |
| Cux-1 | Ⅲb | N | L | E | T | L | I | R | A | N | S | F | V | D | S | K | R | | R |
| 26P4 | Ⅲb | N | L | E | T | L | I | R | A | S | S | F | A | D | N | R | C | | R |
| SD1403 | Ⅱ | N | L | E | S | L | I | R | A | N | S | S | V | D | S | R | C | | R |
| SD15 | Ⅱ | N | L | E | T | S | I | R | A | N | N | F | V | D | S | R | C | | R |
| LF4 | Ⅱ | N | L | E | T | L | I | R | A | N | S | F | V | D | S | R | C | | R |
| HLJ15108 | Ⅱ | N | L | E | T | L | I | R | A | N | S | F | V | D | S | R | C | | R |
| AH4 | Ⅱ | S | L | E | T | L | I | R | A | N | S | F | V | D | S | R | C | | R |
| CAV-EG-14 | Ⅱ | N | L | E | T | L | I | R | A | N | S | F | V | D | S | R | C | | R |
| CAU269-7 | Ⅰ | N | H | E | T | L | V | R | V | N | N | F | V | D | S | R | C | | R |
| 3711 | Ⅰ | N | H | E | | T | L | I | R | V | N | N | F | V | Y | S | R | C | R |
